# Non-Pharmacological Interventions to Manage Type 2 Diabetes in Older Hispanics: A Systematic Review

**DOI:** 10.1093/geroni/igab046.3510

**Published:** 2021-12-17

**Authors:** Edgar Vieira, Kayleigh Sherbutt, Madison Scanlan, Heather Frederick

**Affiliations:** 1 Florida International University, Miami, Florida, United States; 2 Florida international University, Miami, Florida, United States

## Abstract

Type 2 diabetes is a serious public health problem that affects millions of Americans. Hispanics are disproportionately affected and have high incidence of type 2 diabetes. Lifestyle modifications in diet and increased physical activity are recommended in addition to medication. The purpose of this systematic review was to analyze the scientific literature concerning the effects of exercise, nutrition, and combined diet and exercise interventions on type 2 diabetes management in older Hispanics. We searched three databases for studies that included dietary interventions, exercise interventions, or a combination to manage type 2 diabetes in older Hispanics. A total of 653 studies were screened and reviewed, with seven being included in the review. Our findings indicate that physical activity interventions significantly reduce glycosylated hemoglobin, and diet interventions also led to decreased levels of HbA1c. There is a significant effect in HbA1c levels on individuals receiving a combination of diet and exercise interventions compared to control groups. Implementing diet or exercise interventions in older Hispanics with Type 2 diabetes leads to significantly reduced glycosylated hemoglobin levels; the effects of combined diet and exercise interventions were not superior to the effects of single interventions in HbA1c levels. Exercise and diet seem to be effective non-pharmacological interventions to manage type 2 diabetes in older Hispanics, but additional research is needed.

